# Commentary: Role of gut microbiota in infectious and inflammatory diseases

**DOI:** 10.3389/fmicb.2025.1613147

**Published:** 2025-07-18

**Authors:** Gabriela Angélica Martínez-Nava, Alberto López-Reyes, Carlos Pineda

**Affiliations:** ^1^Laboratorio de Gerociencias, Instituto Nacional de Rehabilitación Luis Guillermo Ibarra Ibarra, Tlalpan, Mexico; ^2^Dirección General, Instituto Nacional de Rehabilitación Luis Guillermo Ibarra Ibarra, Tlalpan, Mexico

**Keywords:** gut microbiota, gout, hyperuricemia (HUA), microbiome and dysbiosis, inflammation

## 1 Introduction

The human gut microbiota plays a critical role in maintaining immune homeostasis and modulating inflammatory processes. The review article by Maciel-Fiuza et al. (2023) entitled “*Role of Gut Microbiota in Infectious and Inflammatory Diseases”* provides a valuable overview of the current knowledge in this field, with a focus on autoimmune and infectious diseases. While the article presents a broad and detailed analysis, we noticed that gout, one of the most common forms of inflammatory arthritis, was not discussed. Given the growing body of evidence linking the gut microbiota to gout pathogenesis, we believe its inclusion could further enhance the review and provide a more complete overview on microbiota-associated inflammatory diseases.

### 1.1 Gout: a common and neglected inflammatory arthropathy

Gout is preceded by a prolonged period of hyperuricemia, which triggers the formation and deposition of monosodium urate (MSU) crystals in joints and soft tissues. MSU crystals elicit a potent acute inflammatory response leading to chronic joint damage. Gout affects millions of individuals worldwide, and is associated with a significant morbidity (FitzGerald, 2025). Despite its high prevalence, it is frequently underrepresented in discussions concerning inflammatory diseases related to the microbiome.

### 1.2 Immunometabolic mechanisms in gout

A central component of the inflammatory cascade during gout attacks is the NLRP3 inflammasome, which senses MSU crystals through its LRR domain. NLRP3 inflammasome activation initiates the production of the pro-inflammatory cytokine IL-1β. The ultimate result of this cascade is neutrophil recruitment and the formation of neutrophil extracellular traps (NETs). This process results in joint damage (Liu-Bryan, 2010; Martinon et al., 2006).

**Figure 1:**
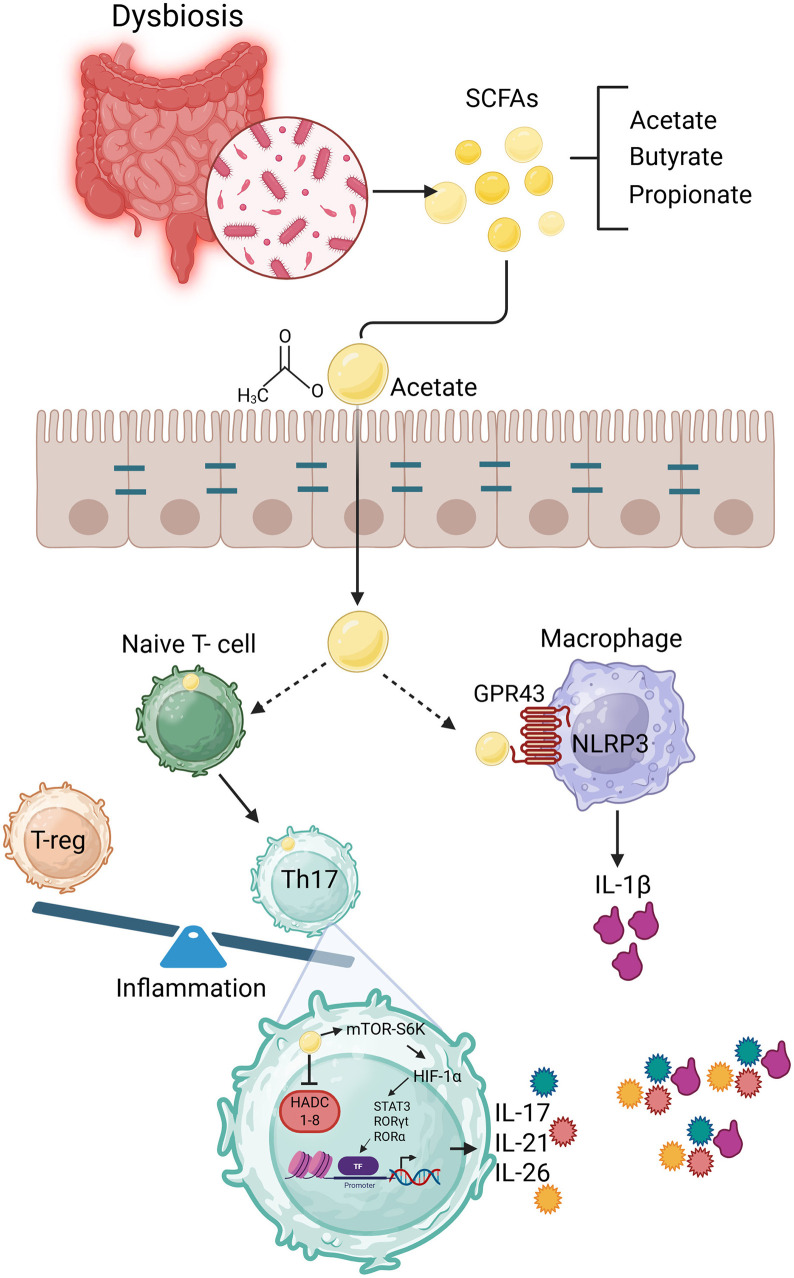
Schematic diagram of the microbiota-SCFA-immune axis in gout pathogenesis. Gut dysbiosis leads to altered SCFA production (notably acetate), which acts through GPR43 to promote IL-1β release in macrophages and Th17 differentiation in naïve T cells, fueling joint inflammation.

Beyond innate immunity, adaptive responses involving T helper 17 (Th17) and regulatory T cells (Tregs) are also implicated in the disease. Th17 cells contribute to sustained inflammation through IL-17 production. On the other hand, Tregs serve to counteract this effect and maintain immune tolerance. An imbalance between these cell types has been associated with exacerbated inflammation in gout (Zi et al., 2024). Notably, microbial-derived metabolites such as short-chain fatty acids (SCFAs) modulate the immune system. Of these SCFAs, acetate has been shown to promote the differentiation of naïve T cells into Th17 cells. This effect involves suppression of histone deacetylases and modulation of the mTOR-S6K pathway (Figure 1). Al together, potentially amplifying inflammatory responses (Park et al., 2015) (Figure 1).

In addition, SCFA-sensing receptors such as GPR43 (FFAR2) and GPR41 (FFAR3), present on neutrophils and macrophages, have emerged as important modulators of the inflammatory response (Maslowski et al., 2009). The recognition of microbiota-derived SCFA, particularly, GPR43 may trigger acute inflammatory arthritis by promoting IL-1β maturation and release through NLRP3 inflammasome activation in macrophage lineage cells (Haslberger and Terkeltaub, 2015).

### 1.3 Gut microbiota–mediated immunometabolic modulation in gout

Emerging evidence demonstrated how the gut microbiota influence both urate metabolism but and the immunometabolic pathways involved in gout (Terkeltaub and Dodd, 2025; Martínez-Nava et al., 2023). Extensive evidence suggests that certain bacteria have developed adaptive mechanisms for purine biosynthesis and salvage, potentially altering systemic urate levels. Additionally, microbial metabolites from the gut microbiota may reach the liver via the portal vein, promoting the synthesis of purinogenic amino acids and urate (Méndez-Salazar and Martínez-Nava, 2022). Notably, fecal microbiota transplants from hyperuricemic (HUA) rats toward germ-free recipients have been shown to increase serum urate levels (Liu et al., 2020).

While most early studies focused on the role of the microbiota in urate metabolism, there is now growing evidence that it also plays a crucial role in modulating the inflammatory response in gout. In a paradigm-shifting study, Vieira et al. (2015) demonstrated that germ-free mice had an impaired inflammatory response to MSU crystals, which was reversed by introducing acetate. This was the first study to highlight the essential role of the gut microbiota in regulating immune response in gout (Vieira et al., 2015).

Studies in in several populations have demonstrated the presence of gut dysbiosis in gout patients, supported by reduced α diversity measures, such as Chao1 and Abundance-base coverage estimator (ACE) indices (Guo et al., 2016; Méndez-Salazar et al., 2021). This dysbiosis is characterized by reduced beneficial taxa (e.g., *Faecalibacterium, Ruminococcus*) and increased pro-inflammatory genera in gout patients (e.g., *Prevotella* and *Bacteroides*) (Martínez-Nava et al., 2023). Moreover, in a metatranscriptomic analysis, Martínez-Nava and colleagues identified at least three orthologues genes (K00161, K00162, and K01621) involved in acetate production pathways that were differentially expressed in the gut microbiota of gout patients compared to normouricemic and asymptomatic hyperuricemic individuals (Martínez-Nava et al., 2025).

Together, these findings suggest that the gut microbiota not only shapes host immunity but also participates in key metabolic and inflammatory processes relevant to gout pathophysiology. This highlights its potential as a therapeutic target in the management of gout.

## 2 Discussion and future directions

The influence of the gut microbiota on systemic immunity has become a focal point in the understanding of chronic inflammatory diseases. While Maciel-Fiuza et al. (2023) address conditions such as rheumatoid arthritis, inflammatory bowel disease, and systemic lupus erythematosus, the omission of gout overlooks a critical intersection of metabolic and inflammatory pathology. Like rheumatoid arthritis, gout is associated with specific dysbiotic signatures and inflammasome activation. However, since gout has a strong connection to diet, metabolism, and microbial metabolites makes it uniquely positioned to benefit from microbiota-centered research.

Current knowledge has unraveled promising strategies for modulating gout associated inflammation. For instance, microbiota-targeted interventions such as probiotics (e.g., *Lactobacillus or Bifidobacterium*); dietary modifications (beyond purine restricted diets to include anti-inflammatory approaches like the Mediterranean diet and diets with high content of fiber); or even SCFA supplementation, specifically butyrate. However, several challenges must be addressed, including individual microbiota variability, safety and efficacy of interventions such as fecal microbiota transplantation: additionally, clinical trials are needed to evaluate the therapeutic impact of such interventions.

Inclusion of gout in future microbiota-focused reviews would help fill this knowledge gap and highlight emerging opportunities for microbiome-based interventions. This is particularly relevant given the increasing prevalence of gout and its strong association with lifestyle factors affecting gut microbial balance. A broader, integrative approach will allow for a more comprehensive understanding of microbiota-driven mechanisms in inflammatory diseases, offering new perspectives for prevention and treatment.

## References

[B1] FitzGerald J. D. (2025). Gout. Ann. Intern. Med. 178, ITC33–ITC48. 10.7326/ANNALS-24-03951 40063960

[B2] Guo Z. Zhang J. Wang Z. Ang K. Y. Huang S. Hou Q.. (2016). Intestinal microbiota distinguish gout patients from healthy humans. Sci Rep. 6:20602. 10.1038/srep20602 26852926 PMC4757479

[B3] Haslberger A. Terkeltaub R. (2015). Editorial: Can GPR43 sensing of short-chain fatty acids unchain inflammasome-driven arthritis? Arthritis Rheumatol. 67, 1419–1423. 10.1002/art.39102 25914362 PMC4446232

[B4] Liu X. Lv Q. Ren H. Gao L. Zhao P. Yang X.. (2020). The altered gut microbiota of high-purine-induced hyperuricemia rats and its correlation with hyperuricemia. PeerJ. 8:e8664. 10.7717/peerj.8664 32185104 PMC7061907

[B5] Liu-Bryan R. (2010). Intracellular innate immunity in gouty arthritis: role of NALP3 inflammasome. Immunol. Cell Biol. 88, 20–23. 10.1038/icb.2009.93 19935768 PMC4337950

[B6] Maciel-Fiuza M. F. Muller G. C. Campos D. M. S. do Socorro Silva Costa P. Peruzzo J. Bonamigo R. R.. (2023). Role of gut microbiota in infectious and inflammatory diseases. Front. Microbiol. 14:1098386. 10.3389/fmicb.2023.1098386 37051522 PMC10083300

[B7] Martínez-Nava G. A. Altamirano-Molina E. Vázquez-Mellado J. Casimiro-Soriguer C. S. Dopazo J. Lozada-Pérez C.. (2025). Metatranscriptomic analysis reveals gut microbiome bacterial genes in pyruvate and amino acid metabolism associated with hyperuricemia and gout in humans. Sci Rep. 15:9981. 10.1038/s41598-025-93899-1 40121243 PMC11929762

[B8] Martínez-Nava G. A. Méndez-Salazar E. O. Vázquez-Mellado J. Zamudio-Cuevas Y. Francisco-Balderas A. Martínez-Flores K.. (2023). The impact of short-chain fatty acid-producing bacteria of the gut microbiota in hyperuricemia and gout diagnosis. Clin. Rheumatol. 42, 203–214. 10.1007/s10067-022-06392-9 36201123

[B9] Martinon F. Pétrilli V. Mayor A. Tardivel A. Tschopp J. (2006). Gout-associated uric acid crystals activate the NALP3 inflammasome. Nature 440, 237–241. 10.1038/nature04516 16407889

[B10] Maslowski K. M. Vieira A. T. Ng A. Kranich J. Sierro F. Yu D.. (2009). Regulation of inflammatory responses by gut microbiota and chemoattractant receptor GPR43. Nature 461, 1282–1286. 10.1038/nature08530 19865172 PMC3256734

[B11] Méndez-Salazar E. O. Martínez-Nava G. A. (2022). Uric acid extrarenal excretion: the gut microbiome as an evident yet understated factor in gout development. Rheumatol Int. 42, 403–412. 10.1007/s00296-021-05007-x 34586473

[B12] Méndez-Salazar E. O. Vázquez-Mellado J. Casimiro-Soriguer C. S. Dopazo J. Çubuk C. Zamudio-Cuevas Y.. (2021). Taxonomic variations in the gut microbiome of gout patients with and without tophi might have a functional impact on urate metabolism. Mol Med. 27:50. 10.1186/s10020-021-00311-5 34030623 PMC8142508

[B13] Park J. Kim M. Kang S. G. Jannasch A. H. Cooper B. Patterson J.. (2015). Short-chain fatty acids induce both effector and regulatory T cells by suppression of histone deacetylases and regulation of the mTOR-S6K pathway. Mucosal Immunol. 8, 80–93. 10.1038/mi.2014.44 24917457 PMC4263689

[B14] Terkeltaub R. Dodd D. (2025). The gut microbiome in hyperuricemia and gout. Arthritis Rheumatol. 19:43118. 10.1002/art.43118 39829115 PMC12276925

[B15] Vieira A. T. Macia L. Galvão I. Martins F. S. Canesso M. C. Amaral F. A.. (2015). A role for gut microbiota and the metabolite-sensing receptor GPR43 in a murine model of gout. Arthritis Rheumatol. 67, 1646–1656. 10.1002/art.39107 25914377

[B16] Zi X. Su R. Su R. Wang H. Li B. Gao C.. (2024). Elevated serum IL-2 and Th17/Treg imbalance are associated with gout. Clin. Exp. Med. 24:9. 10.1007/s10238-023-01253-4 38240927 PMC10799120

